# Society Proceedings

**Published:** 1902-07

**Authors:** 


### SOCIETY PROCEEDINGS.

### REPORT OF THE COMMITTEE ON INTELLIGENCE AND EDUCATION.[1]

By Jacob Helmer, D.V.S., Chairman of Committee,
SCRANTON, PA.

A few years ago in this country instruction in the scientific practice of veterinary medicine was an exception in the literature to which the English-reading and speaking veterinarian had access. Only a few valuable English works were on the market. Yesterday a professional library in the office of a veterinarian was a curiosity; to day the practitioner who does not possess one is liable to the charge of being non-progressive. Without a library for reading and reference it is impossible to keep abreast with the educational advancement around us.

The fact that new books continue to appear shows that there must be an increasing demand, and the more we become a professional body of readers the more and better literature will be placed at our disposal to read.

Among the more recent additions we find:

"Outlines of Clinical Diagnostics of the Internal Diseases of Domestic Animals," by Professor Dr. Bernard Malkmus, of Hanover, Germany, translated by Drs. White and Fisher; "Diseases of Poultry," by Dr. D. E. Salmon, Chief of the Bureau of Animal Industry; "The Sheep," by Dr. William A. Rushworth; "Anatomy of the Cat," an introduction to mammalian anatomy, by Professors J. Reighart and H. S. Jennings, of the University of Michigan; "The Diseases of the Cat," by J. Woodruff Hill; "The American Book of the Dog," edited by G. O. Shields, Coquina, editor of "Recreation;" "Veterinary Materia Medica and Therapeutics," by Kenelm Winslow, of Harvard University; "Compendium of Bacteriology and Blood Serum-therapy," by Professor Paul Jess, Charlottenberg, Germany, translated by Professor Paul Fisher, of the Veterinary Department, Ohio State University; "Text-book of Ophthalmology for Veterinarians," by Professor Moeller, of Berlin, translated by Professor Paul Fisher; "Lameness in Horses," by Professor Joseph Hughes, of the Chicago Veterinary College; "Bovine Obstetrics," by M. G. Debruin, Instructor of Obstetrics at the State Veterinary School, Utrecht, translated by Dr. W. E. A. Wyman; "Synopsis of Professor Quitman's Lectures on Veterinary Materia Medica in the Chicago Veterinary College;" "Synopsis of Professor Baker's Lectures on Theory and Practice of Veterinary Medicine and Surgery in the Chicago Veterinary College;" "Clinical Veterinary Medicine and Surgery," by Professor P. J. Cadiot, Alfort Veterinary School, France, translated by John A. W. Dollar; "Manual of Veterinary Microbiology," translated and edited by Dr. R. R. Dinwiddie, of the Arkansas State University, Agricultural College, and Experiment Station; "Guide to Practical Meat-inspection," by Dr. F. Fishchoeder, Germany, translated

[1] Presented at the annual meeting of the Pennsylvania State Veterinary Medical Association, March, 1902.

by Dr. A. T. Peters, Investigator of Animal Diseases, United States Experiment Station, University of Nebraska; "Methods of the Examination of Milk," compiled by Dr. Paul Sommerfield, of Berlin, translated by Dr. A. T. Peters and R. S. Hiltner, A.M., of the University of Nebraska. A few of the older standard works on milk-inspection are: "Analysis of Milk and Milk-products," Leffman & Beam, published by Blakiston, Son & Co., 1012 Walnut Street, Philadelphia, Pa.; "Outlines of Dairy Bacteriology," by H. L. Russel, sold by the author, address, Madison, Wisconsin; "The Chemistry of Dairying," by Harry Snyder, Chemical Publishing Co., Easton, Pa.; "Testing Milk and its Products," by Farrington and Wall, Mandota Book Co., Madison, Wisconsin; a valuable pamphlet entitled "The Newer Remedies," by Koblentz, published in New York; "Special Surgery of the Upper Air-passages of the Horse," by Professor L. A. Merrilat, of the McKillip Veterinary College, Chicago; "A New Work on Veterinary Medicine," in four volumes, by Professor James Law, Veterinary Department, Cornell University.

The valuable catalogues of standard veterinary publications sent free by William R. Jenkins, 851 and 853 Sixth Avenue, New York, and by Alex. Eger, 34 East Van Buren Street, Chicago, Ill., should be in the hands of every veterinarian in the United States.

But whether a practitioner considers he can or cannot afford all the standard works, he should at least avail himself of the veterinary journals published here. A foreign journal would also be good, as well as a professional periodical on human medicine and surgery.

We should not forget that our American veterinary journals are what we, as a profession, make them. No editor alone can keep a magazine afloat; besides, its intrinsic value will always largely depend upon the co-operation of the profession with its brains and patronage.

In July, 1901, both the scientific and lay world were surprised and shocked by Dr. Koch's announcement at the British Tuberculosis Congress, in substance, that the human family is practically immune to bovine tuberculosis. Should Dr. Koch's conclusions prove true, said fact will not obviate the necessity of inspection of animals and their products and the control work now so rapidly developing in this country. An owner will always desire to weed from his herd any insidious and fatal malady and avoid its reintroduction. Consumers of meat and milk will want only pure and wholesome products. In Germany, where it is the national custom to boil the milk and thoroughly cook the meat, there is no apprehension of disease or uncleanliness. But this prevailing custom there did not prevent tuberculosis from ravaging the herds of Germany until that government was compelled to inaugurate sanitary measures for the control of the spread of the disease.

Because his name is so intimately associated with our knowledge of the subject of tuberculosis, Dr. Koch was the one to raise the question of that most desirable condition, "Immunity of the Human Family to the Bovine Disease." That there is a reasonable chance for such a question there can be no doubt, and in asking it Dr. Koch was honest. It is fortunate, however, that the idea was introduced at the Tuberculosis Congress, because that body challenged Dr. Koch's self-assured position, thus lessening the disintegrating influence upon sanitary work of his practically unsupported conclusions.

It is a credit to the State of Pennsylvania, and a fact of which we should be proud, that our State Live-stock Sanitary Board, with its laboratory for original research, had anticipated the experimental work of Dr. Koch by several years, which results are diametrically opposed to those propounded by Dr. Koch. (See Ravenel's report to the Tuberculosis Congress). Still, more recently, Dr. Ravenel, speaking of the subject, said as follows: "We have had in the laboratory evidence absolutely confirmatory of our former position. We have just published the fourth case of infection of man by bovine bacillus. On the other hand, we have obtained a culture of the tubercle bacillus from the mesenteric gland of a child, which is extremely virulent for cattle, proving either that the human bacillus has at times a virulence for cattle equal to the bovine, or else that the child in question was infected in the first case by the bovine organism. We have had four calves die from inoculation with the human bacillus, and now have a grown animal at the point of death."

During 1900 Drs. Brimhall and Wilson, of the Minnesota State Board of Health, issued a complete and interesting report on a highly contagious and infectious disease of cattle which it had been their privilege to investigate during the summer.

According to the report, ecchymotic spots and hemorrhagic areas were found in nearly all the organs and tissues of the body. Lesions were practically limited to hemorrhagic phenomena. Bacteriological investigation of morbid material resulted in the isolation of the bacillus identified as belonging to the hemorrhagic septicæmic group, of which chicken-cholera, rabbit septicæmia, and swine-plague are members. An infectious disease among wild animals and oxen in Germany has been described and identified as also belonging to the same group.

It appears that Dr. Brimhall and Dr. Wilson were the first in this country to isolate a pathogenic organism from a disease in cattle, which organism resembles and was classified with those producing the hemorrhagic septicæmic group of disease described by German writers.

During the last few years there has appeared in Pennsylvania, chiefly in Carbon and Wayne counties, a disease analogous to that described by Drs. Brimhall and Wilson, of Minnesota.

Descriptions of the symptoms, progress, and termination of the disease, as well as the post-mortem findings, are practically the same in the two places. Thus far the bacteriological work on the disease in this State has been futile, as far as the discovery of the bacillus of Loeffler and Schutz or any other pathogenic organism is concerned. Further, all attempts at direct experimental inoculation from one member of the bovine family to another have failed. The disease appears to be neither contagious nor infectious. It ceases to spread in a few days after the herd has been placed in a field other than that in which the malady originally appeared. Hence it appears to be caused and spread by contaminated food only. The problem has not yet been solved in Pennsylvania.

We are pleased to note the claim made by Copeman, of England, that he has isolated the germ of dog distemper, and has succeeded in producing a vaccine virus which will give immunity to dogs. Prior investigators have made similar claims, and highly vaunted vaccines have been placed upon the market, but as yet we are without the real thing; therefore, let us hope that Copeman has furnished us with an effective means of prevention of the dreadful scourge known as dog distemper. The other most important advances in the bacteriological field during the last year have been in the study of the specific reactions of the blood of different animals. Behring, in Germany, has announced his ability to immunize cattle against tuberculosis. Luclainche, in France, has received a prize from the Academy for the production of a curative and immunizing serum against hog-cholera (most probably our swine-plague); 30,000 animals have been treated, with results ahead of those obtained by diphtheritic serum in human beings.

The recently issued Bulletin No. 79, on Rabies, by Dr. Ravenel, is a most valuable contribution to our literature on this disease. The bulletin excels in that it describes and illustrates the method of diagnosing rabies by means of a microscopical examination of the cervical glands.

By this method a diagnosis can be made in from six to thirty six hours. The idea originated in Europe, but our State Live-stock Sanitary Board was the first to develop and introduce it in this country. When we reflect that the old method by inoculation required several days, and the diagnosis was not more certain, the importance and advantage of the one-day method must be appreciated.

Much research and experimental work has been continued in the laboratory of the State Live-stock Sanitary Board during the past year. The work is of exceeding economic value to the State. It deals with the contagious and infectious diseases of our domestic animals, and furnishes sure means of diagnosis upon which depends rational treatment and prospective eradication of disease. Its work fosters health and protects the lives of the people. Its problems require exact solution, and each result is a stepping stone in the growth and progress of the profession. Recent experiments have been conducted there to demonstrate the varying susceptibility of animals to inoculation tuberculosis and establish the identity of the disease in human and bovine subjects. Again, by what means the disease is extended in stalled herds and the conditions most favorable to rapid expansion; the value of sanitation to cure the malady in the incipient stage and to retard its progress in the more advanced stages; the influence of unsanitary conditions and surroundings upon the spread of the malady in the herd and upon the progress of the disease in victims under unfavorable conditions; the comparative value of curative measures; the development of points in diagnosis and how the tubercle bacillus may enter the milk; practical sanitary measures for the repression of tuberculosis on a large scale as in this State, not only tuberculosis, but anthrax, black leg, and other contagious and infectious maladies against which it aims to fortify farmers free of charge; it has devised a practical method for the treatment and eradication of contagious abortion in herds; tested the value of various disinfectants used in destroying the morbid products and virus of disease; it has demonstrated that the cattle in this country may and do suffer from a lung trouble resembling pleuropneumonia, but caused by a fungus growth in the lungs—the aspergillus fumigatus; it is now at work upon a hitherto unrecognized and fatal malady among cattle in this State resembling anthrax, but which disease does not appear to be contagious. The work of the laboratory is increasing each year in the number of problems presented, the demands of the farmers, and the morbid material sent by veterinarians and others for examination and diagnosis.

When we reflect upon these things we know that the veterinary profession in this State would practically be at sea without such a laboratory. It would be groping in the darkness on scientific problems for want of a search-light, which also prevents retrogression. The laboratory is a school to each intelligent and progressive veterinarian. It means a place for him to send specimens, report the cases of difficult diagnosis, and receive instructions free of charge. It is open daily for his use.

But in addition to the work of the character mentioned, the laboratory is self-sustaining. It has manufactured for use in the State more than enough tuberculin, vaccines, and mallein to defray the cost of its maintenance. Its products for free distribution if purchased in the market would cost the State more than annually the whole expense of the laboratory and the research work done there. The laboratory is supported by a special appropriation from the State. Originally the amount was $12,000 for two years. In 1900 the appropriation was cut to $8000 for two years; last year we asked for $6000 and were granted $5000. Now, suppose that the State grants us $5000 a year for the support of the laboratory, and the latter furnishes products to the amount of more than $5000, what has the laboratory cost the State? Again, consider the value of the original discoveries and the knowledge imparted. But the original object of the State in creating the laboratory was to protect her extensive live-stock interests, which are estimated to suffer six million dollars annually from preventable causes. In the light of this comparison, what does not the State owe the laboratory? Instead of $5000 it would pay the State of Pennsylvania to appropriate for this laboratory $50,000 for its use and expansion to meet the ever-growing need of such work. We feel sure that were this subject properly presented and if the majority of our legislators could be brought to understand the matter thoroughly ample funds would be provided. As it is we do not complain, but it would seem necessary that more money be forthcoming in the future, in order to meet the ever-increasing demand upon the laboratory for scientific work. If each veterinarian would educationally influence his legislative members and induce friends to do so, we could have for our laboratory all we need and want. Just in proportion to the degree of unity and harmony in our ranks will our efforts be characterized by strength and rewarded with success.

The question of what effect mechanical inventions tending to displace the horse will have upon the future of the veterinary profession is one which has caused deep interest and concern everywhere, but especially to the practising veterinarian. Common-sense teaches us that the effort to do the world's work with machinery is not spasmodic or unreasonable. The automobile in its present crude and unsatisfactory form is yet perfect enough to guarantee its permanency. Instead of the so-called fad disappearing, it will continue to increase, and each season will bring forth better machines for less money, and more of them.

No one will deny the fact that the vast majority, if not all veterinary students, enter the profession because they think there is money in it; because the profession is not crowded, and that it will afford them a better livelihood than anything else at hand. If, later in life, a few find that they were adapted to the requirements of the profession, it comes to them as a surprise.

As in all professions, the young veterinarian longs for a city practice, which, in many instances, proves an alluring pitfall. Undoubtedly the cities afford a more lucrative practice, because cities contain many horses in a small area, because there is something to do; besides, there is money with which people are willing to part with in order to have their animals properly cared for.

To veterinarians outside of cities the automobile question has little interest. There will always be as much veterinary work to be done in the country as now. The question will not affect men who fit themselves for State and Government employ and receive a salary. The city veterinarian is the only one who will be injured by the successful advent of the automobile for semi- and heavy-draught purposes.

There can be no longer any question about the application of sufficient power to road vehicles for all purposes. The machines develop sufficient power to climb the highest mountains; then, again, the power cost is merely nominal—a few cents a day. In successful business it is necessary to reduce expenses. If a machine can be utilized that is cheaper than a horse the latter will disappear. Machinery has in the past displaced many kinds of hand work and thrown thousands out of employment. Labor-saving machinery has been a potent agent in the production of hard times.

There is no more sentiment about the use of horses than about the use of man. The moment the horse cannot compete with the machine out he goes. These are facts for the young veterinarian, now on the first round of his professional ladder, to contemplate.

If to-day you should step into France, the birthplace and home of the automobile, you would find their manufacture a booming industry. Exhibition upon exhibition is the thing of the day, and, without doubt, the demand will increase. Thus far the application has been limited practically to small and fancy vehicles for pleasure. Light draught vehicles have not been produced in anything like the same proportion, and but few heavy draught machines are seen; hence we see that the automobile is very popular in France. Its application to semi- and heavy-draught purposes is only a question of time.

But in his struggle for survival against the automobile the horse has a few factors in his favor. The machine, in its present form, must have smooth and dry roads—macadamized roads or their equivalent—the year round. Most machines are so constructed that during cold weather heat is rapidly abstracted from the exposed generators; hence the machine suffers or burns from the increased amount of combustion necessary to supply sufficient power. The automobile in its present form is better adapted to a warm than a cold section, to a dry than a wet locality, to a level than a hilly country. The question of its utility at the present time is one of locality. The horse is adapted to the world, and but few conditions are fatal to him.

The great problem with the inventor is not how to make a machine go, but how to protect it from the disintegrating influences contingent upon bringing it into use to meet all the requirements under favorable conditions at the least possible cost. If automobiles could be run on rails the inventor's dream would be speedily realized; but is not this realization far off? One by one we see the large automobile concerns for heavy draught machines in our large cities going to the wall. On the other hand, the wagon and carriage business is prosperous; and while wagon materials of all kinds are much higher than a few years ago, nearly all manufacturers report a greatly increased trade. The automobile has not thus far displaced the horse for any purpose, because people who have means to purchase machines cannot afford to try to get along without horses.

Concerning matters of legislation, Stiles' Bill No. 51, introduced in the Legislature last spring, was a surprise of no little importance to the veterinary profession in Pennsylvania.

The bill provided for the reopening of the veterinary registry lists throughout the counties of the State for one year, on the ground that there were still men unregistered who were legally entitled to register under the old law, and who, for one reason or another, had never taken advantage of the opportunity.

The defeat of this obnoxious bill was just, and serves to show what may be accomplished by united effort; but while we did exceptionally well and have reason to congratulate ourselves, yet there is reason to believe that we might have done better. Had we contended not for the defeat of this bill but for its amendment only, and had been successful, it would have had the effect of breaking the decision of Judge Schuyler, of Easton, viz.: that you cannot limit the time of registration of one who is legally entitled to register. At the same time the Board of Examiners could have accorded it the power to examine the credentials of those who might present themselves as legally entitled to register according to the decision. It follows that those not able to prove their claim could no longer find protection under the decision, which, by such a method, would have been rendered imperative. Having lost the opportunity afforded last spring, it now behooves the Board to evolve some other plan. As it stands Judge Schuyler's decision is right and just. It is a strong decision, and one that cannot easily be broken. There is an old saying, "If you cannot stem the tide, float gracefully with the stream." Obey the decision, make provisions for such old men as can show they were legally entitled to register under the law. Thus Judge Schuyler's decision may prove a help instead of an obstacle—a hint to clear the field for proper and successful action.

Another interesting bill last spring passed both Houses of the Legislature, and received the signature of the Governor. This ingenious piece of legislation aims to secure a pure and wholesome milk supply for cities of the second class only. In such cities it provides that the sale of milk shall be contingent upon the possession by the seller of a proper license issued by the Department of Health through Councils. The bill empowers Councils to enact legislation requiring all herds that furnish milk to the city to be properly inspected by a veterinarian. It provides both for a physical examination and the tuberculin test. The bill covers the ground of complete inspection at the fountain head—the dairy. It has a few faults, but contains all that is necessary. It is a workable bill, not being too heavy or rigidly exacting. It is to be regretted that the bill does not become the organic law of the entire State. It serves also as a good illustration of what can be done to secure legislation when those desiring it do not ask for the earth.

It is gratifying and portentous that such a widespread interest prevails in the matter of good roads; at least, the work is growing along educational lines.

At the International Good Roads Congress, held in Buffalo in September, 1901, some very valuable papers were read by representatives from a number of the States.

The Committee of the Third Annual State Good Roads Association, which met recently in Albany, reported the following among the recommendations for legislation :

1. That the State be asked to appropriate one million dollars this year for good roads.

2. That improved highways be maintained by the State, the expense to be proportioned between the State, the county, and the town.

3. That a wide tire law be enacted.

4. That a change from the labor to the money system of repairing highways be made compulsory.

The problem of good roads in this country appears to be a most difficult one to solve. All things considered, country roads are but little better in Pennsylvania than they were twenty-five years ago. The labor system, still in vogue, of farmers working out road taxes has been an expensive failure for lack of intelligent road building and sufficient funds with which to build them. No money, no roads.

The idea of the Pennsylvania farmers, expressed through the great organization known as the State Grange, is that a special tax should be levied on all personal and corporate property, and the amount used with that now derived from the taxation of real estate for maintaining and building our roads. Not only that, but personal and corporate property should be taxed for road purposes equal with real estate, mill for mill.

Thus it will be seen how far two great States differ in their ideas of the solution of the good roads problem.

As far as any considerable results can be apprehended from present methods to obtain them, good roads in the United States, such as may be found in Continental Europe, are a Utopian dream. Good roads are not of merely local benefit, but are a necessity to the nation which depends upon them, among other things, for its proper expansion. But unavoidable inter-State obstacles interdict the logic of depending upon the States for the acquisition of good roads for the nation. But these and other obstacles will disappear when the United States Government assumes the responsibility of building and maintaining our public highways. In the meantime let us labor to assist any movement which contemplates the improvement of our roads.

The State Board of Veterinary Medical Examiners during 1901 examined twenty-one candidates for a license of the State, and granted twenty. All graduates must come before the Board. Notices are sent to every graduate from every school in the United States and Canada, where the same are registered from Pennsylvania. Twice each year a letter is sent to each of the sixty-eight prothonotaries in Pennsylvania. The letter reads as follows:

DEAR SIR: In enclosing you a pamphlet copy of the several laws of this State regulating the practice of veterinary science in this Commonwealth, I desire to call your attention to several points upon which prothonotaries have erred and which they have had to appeal to the courts for orders to correct the same on their registers.

1. All graduates of veterinary colleges, as stipulated in Section 1 of the Act of April 11, 1889, were given the privilege of registering as such until the first Monday in September, 1895, as provided for in Section 9 of the

Act of May 16, 1895. After the first Monday in September, 1895, no registrations were admissible save on presentation of a license from this Board.

2. All non-graduates or those not possessing a diploma as provided for in Section 2 of the Act of April 11, 1889, were afforded six months within which they were to make such registration as an "existing practitioner." This period ended October 11, 1889, after which all such registrations were illegal until the passage of the amendment to the law of 1889, on April 29, 1891, when a second period of time was afforded non-graduates until January 1, 1892, after which time all registrations of non-graduates were illegal.

Will you kindly examine your registry and note that all registrations comply with these requirements? Further, that there is no other registration required beyond the one in the county of original registrations; or, in other words, one registration covers the entire State.

Again, that no further registrations can be made in this State save on presentation of a license of this Board with the State seal attached.

Trusting this will receive your careful attention and that you will report at once any irregularities on your registry that this Board may take the necessary steps to correct the same, I am,

Very truly yours,
SECRETARY.

During 1901 there were twenty-two alleged violations of the law investigated by the Board, with three additional ones for 1902 to date. Cases were successfully prosecuted in Schuylkill and Bedford counties. The secretary of the Board has made two trips to York and Columbia to institute proceedings. The case in York is on the list for the April term of court. The one in Columbia has been temporarily postponed in order to secure more evidence. The Board has had names stricken from the registry by order of the court in several counties where the same had been allowed to be improperly placed there by the prothonotaries. All records of the Board are carefully filed and retained in the office of the Secretary, and a detailed record is kept of every violation of the law. The profession is better protected in the State than it ever has been, and the law grows stronger each year.

Without the work of the Board all laws for the protection of the profession in this State instead of being enforced would be a dead letter. As a result, the profession would be in the same position that it occupied previous to the enactment of protective legislation. Thus all illegitimate registrations have been prevented by the influence of the Board upon the prothonotaries of the State, and scores of illegal practitioners have been driven from the field through correspondence. Protection of a profession like ours by laws properly enforced does more to dignify it in the eyes of the people than any other influence. Even the dignity derived from the professional education will not compare with it where the right to practice is not made exclusive.

Along educational lines the work of State Examiners' Boards is no less important. Under their influence college curriculums are altered and improved, courses of study lengthened, higher standing for graduation required, besides an efficient barrier is erected against the propagation of cheap schools.

In Pennsylvania the Board means protection to the interests and also gives elevation to the character of the profession. Therefore, the integrity of the Board should be zealously guarded, to the end that its personnel shall be the best adapted to the purpose.

In closing this subject, would it not be the proper mark of our esteem and appreciation for this Association to frame a resolution thanking Governor Stone for his action in reappointing against political influences an original, also a continuous member, now the worthy and competent Secretary of the Board, Dr. W. Horace Hoskins?

I would suggest for the consideration of this Association the feasibility of having two new committees. A standing committee of three members, to be known as the Committee of Membership. The duty of such a committee would be, first, to obtain the name of every worthy and eligible practising veterinarian in the State; second, to open correspondence with those not already members with a view of bringing them into this Association, and to render a report at each annual meeting showing the extent of the work and what has been accomplished during the year, and all the facts that may be accumulated as a result of the correspondence.

The other committee would be devoted to building up the idea of securing unity and harmony of the profession in Pennsylvania. This committee would not act in reference to members alone, but aim to reach every worthy veterinarian in the State, in order to secure his influence in favor of measures of importance to veterinarians as a professional body.

It is interesting to contemplate that the present year bids fair to excel the past one in the prosperity of the profession's interests. The work of the Bureau of Animal Industry for 1901 shows expansion in almost every direction. It shows that our markets in Europe in the future will depend upon the freedom of our herds from disease; that there has at no previous time been greater danger from imported contagion than now; the present number of quarantine stations are insufficient to afford adequate protection, owing to increased business; the number of cattle and sheep exported has increased over other years; we need no longer to import fine grades of cattle, but should introduce our fine breeding animals to foreign stockmen; prospects for export trade to foreign countries are excellent, but the supply of good stock is scarcely large enough to supply the home demand; the number of cities where meat-inspection is conducted has been increased by 13 per cent. during the year, and the number of carcasses inspected at the time of slaughter by 2,300,000; that there have been a large number of promotions of veterinarians in the Bureau of Animal Industry, and that there is an increased demand for additional inspectors. The report of the chief of the Bureau for 1901 should, on account of its interesting information, be read by every veterinarian.

We note that the last National Horseshow was the most successful ever held in this country; that the good horses have not been as scarce, and, all things considered, as high in the markets as now.

To indicate the status of veterinary education in this country, a few years ago nearly every veterinarian thought he knew everything. Now nobody knows anything. Is not this a potent indication of the progress of intelligence and education? The world owes you nothing. You owe it the influence of your best faculties. Pay the debt you owe and the present will reward you, while coming ages may not cease to bless you.

### LEGISLATION.[1]

By Dr. James W. Sallade, V.S.,
SCHUYLKILL HAVEN, PA.

As one of the Committee on Legislation, I want to report briefly that, as all or most of you are aware, the Stiles bill opening the registration period was defeated by our last Legislature. The push that went to Harrisburg, combined with the individual effort of every member of this Association, had done its work so well that when I went to Harrisburg a second time, before the final vote on the bill, with a willingness to agree to a proposition to allow such as had neglected either by indifference or through ignorance to register in time, but could clearly demonstrate to the State Board their right to do so, had they availed themselves of the opportunity in time to register, the members of the Legislature, I found, had been so thoroughly aroused by their home veterinarians that nothing would suit them but a vote to defeat the entire measure. I was delighted to learn that the profession had so much influence, and make this statement so as to encourage you in concerted action upon legislation.

Many of you who were not placed as I was do not realize your strength.

It developed that the legislator was afraid of the influence of his home veterinarian.

I wish to draw attention to a piece of legislation that I believe would work much good to the profession. I merely touch upon the matter, leaving it open for suggestions, and thereby hope to draw out the full sense of this Association.

I refer to the registration of veterinarians. I would suggest that the next Legislature be asked to alter the law by passing an act requiring registration with the State Board every three or five years at a nominal fee, and repeal the act that requires registration with the prothonotary of each county.

In this manner you would support the hands of your Board. Keep your register clear and require those not associated with us in this Association, but who derive the same benefit from the several acts of the Assembly secured at the hands of the Association, to contribute their efforts to ours in the advancement of the profession.

### REPORT OF THE COMMITTEE ON LEGISLATION.

Since our meeting, one year ago, we have closed a session of our Legislature in Pennsylvania, which I have no doubt you are familiar with. Before this body there were several bills that we were interested in for the welfare of our State Live stock Sanitary Board, and one bill that we were most especially and deeply concerned in. You will recall our adjournment one year ago under the necessity of going to our State capital, there to vigorously protest before a committee of the House against the passage of a Senate bill known as the Stiles bill, destined to reopen the registration of non-graduates. Those of you who were among the body that personally

[1] Presented at the Annual Meeting of the Pennsylvania State Veterinary Medical Association, March 4 and 5, 1902.

protested against the measure will remember the favorable assurances from the Public Health Committee that this bill would not be favorably recommended, and recall the fact that they returned the bill to the House with a favorable recommendation, all at the dictation of certain powerful influences who were using this as well as other pernicious measures for ulterior purposes. Realizing the unwarranted and unmerited strength back of this measure from that hour on until the defeat of this vicious proposition, we were compelled to wage daily and hourly warfare against this bill and the calling of every veterinarian from Lake Erie to the Delaware to personally use his influence at home and at Harrisburg against this proposed dangerous legislation, and nothing in the world saved us from its evil influences but the almost unanimous flood of protests by letter, by wire, phone, and personal visits filed with our legislators in the State capital. This bill was the greatest test of the association worth and professional union, and a triumphant trial of our combined strength. Our present laws have undoubtedly worked some hardships, but these are the great exceptions, and should not be counted against the great benefits derived by our people in better educated and trained veterinarians.

Two other measures of interest to us as veterinarians were passed at the last session of the State Legislature, one of which will greatly contribute to the efficiency of the work of the State Live-stock Sanitary Board. In compelling the proper disposal of the carcasses of animals dying with infectious and contagious diseases much will be saved in deterring the spread of these diseases as well as preventing their becoming fixed on farms and grazing grounds.

A second bill was passed whereby the owners of horses and cattle, as well as sheep, may be compensated for the loss of these animals from the dog-tax fund where they have died or been destroyed as suffering from "rabies." This law, it is thought, will contribute to the more thorough collection of dog taxes, the destroying of homeless dogs and wandering curs, and be a source of relief to those who suffer losses through this malady. It may require some modification, in that all such losses shall be certified to by the State Live-stock Sanitary Board to avoid possible unjust losses.

Respectfully submitted,

W. Horace Hoskins.

### PENNSYLVANIA STATE VETERINARY MEDICAL ASSOCIATION.

### Committees, 1902–03.

*Committee on Legislation:* George Jobson, Chairman, Franklin; W. L. Kooker, 1116 Wallace St., Philadelphia; J. W. Sallade, Schuylkill Haven; W. H. Ridge, Trevose; H. S. Jackson, Sewickley; James C. McNeil, Department of Public Safety, Pittsburg; E. C. Porter, New Castle.

*Committee on Intelligence and Education:* H. B. Felton, Chairman, Olney; B. F. Bachman, 1 Alpha Terrace, Beatty St., Pittsburg; A. O. Cawley, Arch St., near Broadway, Milton; S. D. Larzelere, Jenkintown; Otto G. Noack, corner of Sixth and Cherry Sts., Reading; Charles Williams, 1321 Thompson St., Philadelphia; Edwin Hogg, 11 W. Canal St., Wilkesbarre.

*Committee on Sanitary Science and Police:* John E. Spindler, Chairman,

524 Duquesne Way, Pittsburg; J. R. Courtright, Clark's Green; E. E. Biddle, New Castle; C. W. Holley, Muncy; R. M. Phelan, Sharon; M. Moriarty, Gettysburg; M. J. Chrisman, Sugar Grove.

*Committee on Animal Husbandry:* S. J. J. Harger, Chairman, 205 N. Twentieth St., Philadelphia; J. L. Bradley, Mercersburg; W. H. Fry, Pine Grove Mills; J. B Irons, 144–46 W. Fourteenth St., Erie; F. F. Hoffman, Brookville; E E. Tower, Montrose; John F. Vandergrift, Newtown.

*Committee on Milk:* J. C. Helmer, Chairman, Scranton; J. M. Carter, 1725 N. Fifteenth St., Philadelphia; C C. McLean, Meadville; M. E. Conard, West Grove; Edward Seidel, Carlisle; J. C. Lacock, 38 N. Diamond St., Allegheny; E. T. Cornman, York.

*Committee on Press:* Benjamin Underhill, Chairman, Media; U. S. G. Bieber, Kutztown; W. G. Boyd, 26 S. Diamond St., Allegheny; C. Z. Laberge, Beaver Falls; J. Z. Tintzman, 335 E Girard Ave., Philadelphia; C. F. Keiter, Elizabethville; W. L. Phillip, Reading.

*Committee on Meat-inspection:* E. W. Newcomer, Chairman, Mt. Joy; George H. Dunn, 733 Arken Ave., Pittsburg; H. W. Turner, Lahaska; Henry Mayer, 4722 Liberty Ave., Pittsburg; C. T. Goentner, Bryn Mawr; Joseph Johnson, West Grove; G. K. Swank, East Mauch Chunk.

*Committee on Army Legislation:* W. Horace Hoskins, Chairman, 3452 Ludlow St, Philadelphia; James A. Waugh, 1 Robinson St., corner of Fifth Ave., Pittsburg; Leonard Pearson, Thirty-sixth and Spruce Sts., Philadelphia; Thomas B. Rayner, Highland Ave., Chestnut Hill, Philadelphia; J. H. Oyler, 19 S. Fourth St., Harrisburg.

DELEGATES.

American: S. J. Foelker, 119 S Seventh St, Allentown, Pa.; N. Rectenwald, 89 Washington Ave, Pittsburg, Pa.; J. C. Michener, Colmar, Pa.

New Jersey: C. J. Marshall, 2004 Pine St., Philadelphia, Pa.; Charles Williams, 1321 Thompson St., Philadelphia, Pa; W. S. Kooker, 1116 Wallace St., Philadelphia, Pa.

New York: James B. Rayner, 135 E. Gay St., West Chester, Pa.; W. B. E. Miller, 333 Market St., Camden, N. J.; James C. McNeil, Department of Public Safety, Pittsburg, Pa.

Keystone: Francis Bridge, 228 N. Fifty-third St., Philadelphia, Pa.; H. P. Eves, 504 W. Ninth St., Wilmington, Del.; Charles Lintz, 1003 Madison St., Chester, Pa.

Virginia: James Mahaffy, Jackson and Ninth Sts., Wilmington, Del.; A. N. Lushington, 1101 Fifth Ave, Lynchburg, Pa.; Joseph F. Ross, Frankford Ave. and Allen Sts., Frankford, Philadelphia, Pa.

Ohio: John Bryce, Fifth and French Sts., Erie, Pa.; David Waugh, 813 Forbes St., Pittsburg, Pa.; W. A. Meredith, Corry, Pa.

### SCHUYLKILL VALLEY VETERINARY MEDICAL ASSOCIATION.

THE annual meeting of this Association was held at the Park Hotel at Pottsville, June 25, 1902. Dr. O. G. Noack, the President, called the gathering to order at 11 A.M. The following members responded to roll-call: Drs. Longacre, Shenandoah; Wehr, Denver; Bieber, Kutztown; Noack, Reading; Huyett, Wernersville; McCarthy, Pottsville; Kauffman,

Ashland, and Newhard, Ashland. A number of visitors were present. The President delivered his address, which was replete with interesting and instructive thoughts.

The election for the ensuing year resulted: President, Dr. O. G. Noack, Reading; Vice-President, Dr. G. A. Wehr, Denver; Recording Secretary, Dr. U. S. G. Bieber, Kutztown; Corresponding Secretary, Dr. W. G. Huyett, Wernersville; Treasurer, Dr. F. McCarthy, Pottsville. Trustees, Dr. Fred. Schneider, Philadelphia; Dr. I. C. Newhard, Ashland; Dr. D. R. Kohler, Boyertown. Committee on Intelligence and Education, Dr. W. S. Longacre, Mantz; Dr. A. R. Potteiger, Selinsgrove; Dr. Eli Snyder, Orwigsburg; Dr. H. F. Kauffman, Ashland. A motion was then made and carried to adjourn for luncheon, which was done. At 1 P.M. the meeting was again called to order by the President, when a number of essays and papers were presented and ably discussed by all the members present. One paper of especial interest was "The Value of Antitoxins," by Dr. F. Schneider, who was unavoidably absent. The subject was discussed and some interesting experiences were related.

Half an hour was allotted to the payment of dues, after which the subject of collections from those in arrears was referred to, and the Secretary was instructed to correspond with those in arrears and report at the semi-annual meeting. The Secretary and Treasurer's reports were audited, showing the Association to be in a flourishing condition.

Letters regretting absence from the meeting were read from Drs. Pearson and Kohler, of Philadelphia and Boyertown, respectively.

A vote of thanks was tendered Dr. Leonard Pearson, State Veterinarian, for his kindness in sending the valuable circulars on "The Intercommunicability of Human and Bovine Tuberculosis," by Dr. M. P. Ravenel, to the President, who distributed one to each member. The Secretary was instructed to mail a copy to each absent member and to other veterinarians. The subject of registration was again brought up, and much confidence was expressed that it will become a law in the near future. The gathering adjourned, to meet at the call of the Secretary. The semi-annual meeting will be held in the Board of Trade Building, at Reading, in December.

### ALUMNI SOCIETY OF THE VETERINARY DEPARTMENT OF THE UNIVERSITY OF PENNSYLVANIA.

THE regular annual meeting of the Alumni Society of the Veterinary Department of the University of Pennsylvania was held in Houston Hall, June 18, 1902, with the following alumni present:

C. J. Marshall, 1894; S. J. J. Harger, 1897; Casper Garrett, 1888; A. F. Schreiber, 1888; Charles Lintz, 1887; W. R. Andrews, 1900; G. W. Homer, 1900; Charles E. Magill, 1893; J. Alvin James, 1893; H. D. Martien, 1896; Frederick Stehle, 1901; Edgar W. Powell, 1900; T. S. Carlisle, 1901; J. D. Houldsworth, 1902; F. H. Bradley, 1902; Fred. Weitzel, 1902; A. A. Harmon, 1902; W. J. Storm, 1897; H. Feigenbaum, 1902; H. Baker, 1902; S. C. Babson, 1902; Oscar F. Stearns, 1902; John W. Adams, 1892; Leonard Pearson, 1890; S. J. Cole, 1902; Samuel Burrows, 1902; E. M. Ranck, 1897; W. Horace Hoskins, D.V.S., American Veterinary College, New York, and Thomas B. Rayner, Philadelphia College of Veterinary Surgeons.

The minutes of the previous meeting were read and approved, after which we had reports of various officers and committees.

The Library Committee, under chairmanship of Dr. Leonard Pearson, brought to our attention the necessity of libraries in veterinary colleges, and through his efforts a committee was formed to see if the library of the late Dr. Rush Shippen Huidekoper could not be bought. It was the intention of this committee to have a number of contributions made, so that this library could be procured for the veterinary department of the University of Pennsylvania.

Dr. Rayner was among those who were approached on this subject, and out of his generous nature he willingly assumed the responsibility of purchasing the library and presented it to the veterinary department. The only restriction Dr. Rayner has placed on this purchase is that it shall be available for all veterinarians throughout the country, and we are glad to say that since it has been placed in the library building of the University of Pennsylvania it will be accessible at all times, as this rule prevails there.

This library is presented as a memoir to Dr. Rayner's late son, Moncure R. Rayner, who died while pursuing his course in our department.

Dr. Adams, of the same committee, in making a report under the same heading, said: "It is the intention, as soon as we have a fireproof building, to have those books removed to the veterinary department proper." He expressed his gratitude in a very pleasing manner to Dr. Rayner, and said that he hoped we would show our appreciation by our assuming the task of adding to this munificent gift.

Dr. Harger, in making a few remarks in reporting on the same committee, said "that Dr. Rayner wishes to improve the status of the profession from his extreme love for the same." Dr. Harger, who knew his son so very well, who took a great interest in his studies, referred to the fact that had he not been taken from us he would probably be one of the shining alumni of our department now.

[The further reference to the report of this gift will be found under the head of resolutions.]

This report was accepted as above and ordered to be spread upon the minutes.

The report of the other Library Committee was not available. This committee, having been recently appointed, did not have time to prepare a report.

The Secretary and Treasurer reported the financial condition of the Society, which shows it to be in a prosperous state. A number of the graduating class showed their interest in the Association by enrolling their names on the membership list.

Following this was the annual election of officers, which resulted as follows: President, A. F. Schreiber; Secretary and Treasurer, E. M. Ranck; Historian, S. J. J. Harger; Executive Committee, Charles Williams, S. J. J. Harger, and Leonard Pearson.

Under the head of new business, resolutions were adopted as follows:

*Whereas*, It has pleased Divine Providence to remove from our midst our late fellow-alumnus and esteemed friend and colleague, Dr. Frank T. Shannon, whose integrity, noble impulses, and professional ability were appreciated by us all, and whose endeavors were always exerted for the welfare and prosperity of the veterinary profession, be it, therefore,

*Resolved*, That this Association sincerely regrets his loss so early in his useful career, and extends to his parents its sympathy and condolence in their great loss; and be it further

*Resolved*, That these resolutions be spread upon the records of this Association, and a copy thereof be transmitted to the parents of our deceased friend and to each of the veterinary journals in the United States.

*Whereas*, In response to the immutable laws of Almighty God, there has been called from the strife and turmoil of earthly cares an alumnus of this Society, Dr. James Beatty, again we are reminded of the uncertainty of life and the certainty of death. We mourn his early demise, not more in response to a sympathetic chord of sadness in our hearts than because we realize the blighting of a bright prospect of future development into strong manhood, a useful member of society, and a benefit to his profession. His integrity of purpose, purity of character, and loyalty to his many friends endeared him to this Society and made him an associate worthy of emulation. Endowed with a cheerful disposition and a warm, sympathetic nature, he wound about all our hearts the tendrils of his love and affection. It may be truly said of him that he was a wise and valued counsellor in prosperity and a sincere friend in adversity. The members of this Society, and those to whom his qualifications were best known and appreciated, can only bathe his memory in their tears and lay upon his resting-place the wreath of their affection; therefore, be it

*Resolved*, That by the death of Dr. Beatty this Society has sustained a loss of one of its brightest, most useful, and beloved members, and the University of Pennsylvania a warm supporter and one of its representative men; that the ranks of the profession for the practice of which he had prepared himself will feel the loss of one worthy of the highest honors possible of attainment, and that society at large is deprived of the influence he might have exerted and the good he might have done.

*Resolved*, That a copy of these resolutions be spread upon the records of this Society and that they be published in the *Alumni Register*.

*Whereas*, Through the generosity of Dr. Thomas B. Rayner, of Chestnut Hill, the large and valuable library of the late Dr. Rush Shippen Huidekoper has been purchased and presented to the Veterinary Department of the University of Pennsylvania, as a perpetual memorial to his son, Moncure Robinson Rayner, Class of '96;

*Resolved*, That we, the Alumni of the Veterinary Department of the University, are profoundly grateful for this munificent gift, and do pledge ourselves to do all in our power to enlarge and render efficient the Rayner Library; be it, therefore,

*Resolved*, That Dr. Thomas B. Rayner be elected an Honorary Member of the Alumni Society of the Veterinary Department of the University of Pennsylvania; be it further

*Resolved*, That an appropriate tablet, inscribed with the name of the donor and his magnificent gift, be placed in the library room of the new Veterinary Building.

A plan was proposed for the perusal of the members present to establish a fund for the library by holding shares of stock in a building association. This was thoroughly discussed by members present who are members of various building associations, and also by Dr. Hoskins, who has been a director of one for a number of years.

A motion to have the Library Committee given power of attorney to formulate plans to start such a fund, to work in connection with the Executive Committee, was unanimously adopted.

W. Horace Hoskins, D.V.S., Alexander Glass, V.S., and John Marshall, M.D., were elected Honorary Members.

We next listened to an excellent address from Dr. Leonard Pearson for the proposed plans for the new buildings of the veterinary department, which we have every reason to expect to be completed in a very short time.

The following committees were appointed by the incoming President for the ensuing year: Library Committee, Drs. Adams, Pearson, and Harger; Special Library Committee, Drs. Felton, Repp, and Mohler.

After a general smoke, this being a smoker instead of a banquet, and listening to various remarks from the alumni present, the Association adjourned.

E. M. RANCK,
Secretary-Treasurer.

---

Secretary Ranck, of the Pennsylvania State Veterinary Medical Association, is out with a preliminary notice of the semi-annual State meeting on September 16, 1902. A number of good papers are assured for the programme.

THE Missouri Veterinary Medical Association will hold their eleventh annual meeting in St. Louis, August 18th and 19th. The programme embraces a number of very interesting subjects, including a surgical and dental clinic by the members resident of St. Louis, and an exhibition and demonstration of a modern rocker operating-table. The reception committee, Dr. C. W. Crowley, chairman, has associated with him the most active and prominent veterinarians in St. Louis, which assures a cordial reception and a pleasant entertainment.

---

KANSAS CITY VETERINARY COLLEGE. The twelfth annual announcement for the college year 1902-03 has been received from this college. The following changes are noted: In faculty, Warren J. Fretz, D.V.S., Physical Diagnosis and Examination for Soundness, in place of Henry C. Babcock, V.S., M.D.; Arthur Trickett, D.V.S., Comparative Anatomy, in place of Charles M. Davies, M.D., V.S. Added: Silas L. Brooking, M.D., V.S., on Hygiene. Dropped: Charles J. Sihler, D.V.S., on Milk and Dairy Inspection; Robert Jay, D.V.M., on Diagnosis and Treatment of Lameness; W. H. Tuck, Demonstrator of Bacteriology. Added as text books: *Materia Medica and Therapeutics*, Winslow; *Lameness*, Wyman; as books of reference: *Pathology*, Ziegler; *Feeds and Feeding*, Armsby.

